# Current Surgical Indications for Non-Small-Cell Lung Cancer

**DOI:** 10.3390/cancers14051263

**Published:** 2022-02-28

**Authors:** Nathaniel Deboever, Kyle G. Mitchell, Hope A. Feldman, Tina Cascone, Boris Sepesi

**Affiliations:** 1 Department of Thoracic and Cardiovascular Surgery, The University of Texas MD Anderson Cancer Center, Houston, TX 77030, USA; ndeboever@mdanderson.org (N.D.); kgmitcchell@mdanderson.org (K.G.M.); hfeldman@mdanderson.org (H.A.F.); 2 Department of Thoracic/Head and Neck Medical Oncology, The University of Texas MD Anderson Cancer Center, Houston, TX 77030, USA; tcascone@mdanderson.org

**Keywords:** non-small-cell lung cancer, lobectomy, pneumonectomy, sublobar resection, surgery, enhanced recovery pathways, thoracoscopy, video-assisted thoracoscopic surgery, robotic-assisted thoracic surgery

## Abstract

**Simple Summary:**

The management strategy for the treatment of non-small-cell lung cancer (NSCLC) has been transformed by our improved understanding of the cancer biology and concomitant development of novel systemic therapies. Complete surgical resection of NSCLC continues to offer the best chance for cure or local and regional disease control, and with improvements in minimally invasive techniques and enhanced recovery, the morbidity associated with surgical resection has been reduced. Patient-centered multi-disciplinary discussions that consider surgical therapy are associated with improved outcomes. Provided with promising novel therapeutic modalities including immune checkpoint inhibitors with or without chemotherapy, stereotactic radiotherapy, and targeted systemic therapies, indications for surgery continue to evolve and have expanded to include selected patients with advanced and metastatic disease.

**Abstract:**

With recent strides made within the field of thoracic oncology, the management of NSCLC is evolving rapidly. Careful patient selection and timing of multi-modality therapy to permit the optimization of therapeutic benefit must be pursued. While chemotherapy and radiotherapy continue to have a role in the management of lung cancer, surgical therapy remains an essential component of lung cancer treatment in early, locally and regionally advanced, as well as in selected, cases of metastatic disease. Recent and most impactful advances in the treatment of lung cancer relate to the advent of immunotherapy and targeted therapy, molecular profiling, and predictive biomarker discovery. Many of these systemic therapies are a part of the standard of care in metastatic NSCLC, and their indications are expanding towards surgically operable lung cancer to improve survival outcomes. Numerous completed and ongoing clinical trials in the surgically operable NSCLC speak to the interest and importance of the multi-modality therapy even in earlier stages of NSCLC. In this review, we focus on the current standard of care indications for surgical therapy in stage I-IV NSCLC as well as on the anticipated future direction of multi-disciplinary lung cancer therapy.

## 1. Introduction

Lung cancer affects an estimated 2 million new patients each year and is associated with 1.76 million deaths per year making it the leading cause of cancer-related death in the world [1]. Surgical management of lung cancer remains the modality most likely to result in curative outcomes while providing locoregional disease control. Surgical techniques and approaches have been continuously improving along with the vast array of other compelling treatment modalities developed for lung cancer management. This has enabled more patients to undergo operations while minimizing post-operative morbidity and mortality. The cohesive collaboration amongst multiple disciplines has led to advancement in the comprehensive management of patients with NSCLC.

## 2. Principles of Surgical Therapy for Lung Cancer

The aims of surgical therapy for NSCLC are to perform a safe and effective operation in order to achieve complete resection with negative margins and adequate clearance of lymph node basins that are at risk or involved and to return a patient to a functional state to either undergo additional therapy or return to his or her pre-operative activities. This overarching principle incorporates a patient-centered approach, with critical and intricate patient selection, leading to therapeutic options that optimize oncologic benefit and minimize risks of complications while considering goals of care set by the patient. Pulmonary function tests and predicted post-operative values are used to identify the ability of a patient to undergo resection safely and are combined with cardiovascular status and additional deleterious comorbidities that may represent contraindications.

The choice of procedure and approach comes with the decision to operate. There are trials investigating the outcomes from sublobar resections compared to lobectomies or pneumonectomies, providing data specifically regarding oncological outcomes relative to the size of the tumor and nodal status, setting the lobectomy as the standard and most common oncologic resection [2] while retrospective work highlights that sublobar resections can be oncologically sufficient in a highly selected cohort [3,4,5].

Peri-operative mortality and morbidity continue to improve with the propagation of enhanced recovery after thoracic surgery (ERATS) pathways. The use of post-operative early ambulation, multimodal opioid-sparing analgesia [6], and reduction of surgical stress has led to improved post-operative outcomes including pain control [7], decreased length of stay, and decreased pulmonary and cardiac morbidity during open operations [8,9]. Most importantly, implementation of the ERATS pathways has facilitated the delivery of adjuvant chemotherapy. By promoting a more rapid return to baseline function, ERATS has enabled patients to resume systemic therapy more quickly and facilitates the completion of full four cycles of therapy [10]. These concepts are important especially with the approval of osimertinib [11] and atezolizumab [12] as adjuvants to surgery and chemotherapy in stage IB-IIIA NSCLC.

Mediastinal lymph node sampling during index operation compared to complete dissection also remains a source of discussion. Comprehensive nodal assessment is integral to the principles of surgical therapy for lung cancer. Clearance of at-risk lymph nodes is a cornerstone of optimizing survival benefit and depends on mediastinal nodal disease status. While the evidence remains equivocal, a large randomized controlled trial (ACO-SOG Z0030) highlighted that in the event that systematic mediastinal and hilar lymph node sampling [13] is negative, completion of mediastinal lymph node dissection did not improve survival in patients with N0 or nonhilar N1, T1, or T2 NSCLC [14]. Whether nodal dissection of clinically positive mediastinal lymph nodes improves survival remains unanswered; however, authors recommend complete ipsilateral mediastinal nodal dissection in this setting to enhance regional disease control.

## 3. Staging and Classification of Lung Cancer

The TNM (tumor, node, metastasis) staging schema, originating in the 1970s [15], has continued to evolve along with our knowledge of lung cancer. The current eighth edition of the American Joint Committee on Cancer’s TNM lung cancer classification was introduced in 2017 [16]. Stage 0 encompasses all NSCLCs with a tumor that has not invaded the submucosal layer. Stage Ia relates to node-negative tumors that are less than 3 cm, while stage Ib relates to tumors that measure up to 4 cm. Stage II NSCLC relates to tumors that are less than 5 cm with nodal spread or less than 7 cm without nodal spread. Stage III comprises larger tumors and is divided into surgically resectable or unresectable. Stage IV NSCLC is routinely unresectable and has spread distally with metastases [16,17]. Treatment is often determined by the stage of disease at the time of diagnosis, with surgery considered an appropriate adjunct to multimodal therapy for stages I–III and oligometastatic disease.

## 4. Surgical Indication by Stage

### 4.1. Stage I

#### 4.1.1. Stage Ia

Patient-centered treatment is the cornerstone of lung cancer surgical management, and medically operable patients with stage 1 lung cancer should be considered for curative-intent pulmonary resection. Numerous studies have concluded that surgical management of this patient population is the standard of care and provides superior outcomes and locoregional control compared to other modalities, in part due to the benefits associated with mediastinal lymph node dissection both for further diagnostic and curative purposes [18,19]. Following the decision to operate on this patient population, the extent of the procedure can be a source of discussion [20].

Multiple investigations have shown that in the case of tumors less than 2 cm in size, a segmentectomy can lead to oncologically sufficient outcomes [21,22,23,24] and lung-cancer-specific survival [25] without any difference in perioperative mortality or morbidity [3]. As such, segmentectomy should be strongly considered in this population as outcomes are comparable to lobectomy.

For tumors 2.1–3 cm -in size, lobectomy remains the standard of care [21,26] while segmentectomy can be considered as a recent investigation established similarity in oncologic and overall outcomes between segmentectomy and lobectomy for patients without nodal disease [27]. A large phase 3 clinical trial (NCT00499330) due to be completed in 2024 will provide further evidence regarding the optimal surgical approach (lobectomy versus segmentectomy or wedge resection) for management of stage 1 lung cancer [28] (Table 1). The decision whether to perform a resection with minimally invasive techniques such as video-assisted thoracoscopic surgery (VATS), robotic-assisted thoracoscopic surgery (RATS), or in an open manner remains associated with patient-centered factors [29,30] considering that both open or minimally invasive approaches show similar oncologic outcomes, with VATS being associated with longer operative time [31], but both minimally invasive approaches leading to shorter hospital stays [32].

The best alternative to surgical resection for stage I NSCLC is stereotactic ablative radiotherapy (SABR). Previous work subjected to ongoing discussion concluded that SABR showed non-inferiority to minimally invasive lobectomy with mediastinal lymph node dissection with similar 3-year overall survival between propensity-matched cohorts following multidisciplinary discussion and patient preference; however, these trials were slow to accrue and performed their analysis early [33,34].

#### 4.1.2. Stage Ib

Patients with stage 1b disease will have tumors larger than 3 cm but smaller than 4 cm [17]. These patients should undergo primary tumor resection followed by tumor profiling [35], specifically to investigate mutations including those related to the epidermal growth factor receptor (EGFR) [11,35], which then permits consideration for targeted therapy.

Patients with tumors between 3 and 4 cm will also benefit from a primary operation, the extent of which deserves deliberation. The options consist again of sublobar or lobar resection. A large retrospective study has shown that patients undergoing lobectomies for tumors between 2 and 5 cm were more likely to have >10 lymph nodes removed which was associated with improved survival and cancer-specific mortality [36]. Despite slightly larger tumors than those seen in stage 1a, this cohort continues to benefit from minimally invasive resections and its associated decreased morbidity compared to open approaches [37].

Therapy utilizing tyrosine kinase inhibitors, specifically osimertinib, has shown superiority in patients with EGFR mutations, with prolonged disease-free survival [11], a benefit that persisted on subgroup analysis of patients with stage 1b disease. Considering the use of neoadjuvant chemotherapy, despite being associated with downstaging in some patients, was not associated with having any effect on oncologic surgical outcomes or overall survival in patients with stage 1b NSCLC [38,39]. It is debated whether patients with stage 1b disease will benefit from adjuvant chemotherapy according to revised analyses from the Cancer and Leukemia Group B (CALGB) 9633 Trial indicating only a trend toward survival benefit in select patients with stage IB disease (tumors ≥ 4 cm in size) [40,41].

### 4.2. Stage II

Patients suffering from stage 2 lung cancer will benefit from surgical resection as well, with aims of cure and locoregional control. However, patients with stage II also need systemic therapy, and this stage meets inclusion criteria for all ongoing neoadjuvant and adjuvant clinical trials as well as the standard of care adjuvant chemotherapy plus targeted or immunotherapy. Special attention must be placed on multidisciplinary discussions and multimodal protocols as the evidence for stage 2 disease is scarce due to a paucity of patients diagnosed at this stage. There are clinical trials investigating the optimal strategy to manage these patients using the currently available modalities, bolstering that the use of adjuvant chemotherapy has shown benefits in this cohort [42] even in patients with completely resected tumors [43], while the use of post-operative radiotherapy (PORT) is associated with benefits in patients with incompletely resected stage 2 disease [44] and decreased mediastinal relapse without affecting disease-free survival (DFS) in patients with N2 involvement and complete resection [45].

Given the paucity of evidence surrounding the optimal extent of surgical resection, patient-centered decisions must be made. In a recent retrospective study that included over 60 patients with stage 2 lung cancer within a larger cohort of patients with stage 1 lung cancer, early results show that long-term outcomes were similar between sublobar resections and lobectomies [46]; however, these results merit further conscientious investigation following full publication of their results. In this patient population, mediastinal lymph node dissection must occur and is associated with a benefit in locoregional disease control when compared to mediastinal lymph node sampling only, with improved 5-year survival [47,48] and without any added post-operative mortality [49]. Authors favor lobectomy with mediastinal node dissection in this setting.

### 4.3. Novel Complementary Therapies for Resectable Stage II and III NSCLC

Immunotherapy has dramatically progressed in the last decade and has offered compelling neoadjuvant opportunities to synergistically enhance major pathologic response [50,51]. Blockade of targets at the immune checkpoint has led to comprehensive research investigating its associated benefits [52]. Immune checkpoint inhibitors (ICIs) including those that block programmed cell death proteins (PD-1) have been associated with antitumor immune responses. A new paradigm comprising neoadjuvant chemotherapy combined with immunotherapy appears to show advantageous survival outcomes in NSCLC. Neoadjuvant use of nivolumab was feasible and associated with major pathological response in 45% of patients with resectable NSCLC in the first pilot study [50]. In a phase 1b trial, neoadjuvant sintilimab induced a major pathologic response in 40.5% of Asian patients with resectable NSCLC [53]. The NEOSTAR phase 2 randomized trial showed that neoadjuvant nivolumab plus ipilimumab, a CTLA-4 inhibitor, was promising for further testing as compared to historical controls of neoadjuvant platinum-based chemotherapy in patients with resectable NSCLC, as determined by major pathological response [51]. The Neoadjuvant Chemotherapy and Nivolumab in Resectable NSCLC (NADIM) phase II trial showed that combining neoadjuvant platinum-based chemotherapy with nivolumab and with 12 months of adjuvant nivolumab in patients with stage III resectable disease led to a 24-month progression-free and overall survival of 77% and 90%, respectively [54]. The robust neoadjuvant management strategy in the resected cohort was associated with a high rate of major pathological response (83%), and 63% benefited from complete pathological response [54]. The use of neoadjuvant PD-L1 inhibitor atezolizumab, in resectable disease, has led to a 20% major pathological response rate [55]. This effect remained present when atezolizumab was used in combination with neoadjuvant chemotherapy with a promising rate of MPR without any surgical resection compromises [12].

Durvalumab was shown to lead to high major pathologic response rate and a 1-year event-free-survival rate of 73% when used in the perioperative setting with concurrent neoadjuvant chemotherapy in the phase 2 SAKK 16/14 study [56].

The phase 3 trial Checkmate 816, further investigating the use of nivolumab plus chemotherapy versus chemotherapy alone, showed benefits associated with complete pathological response [57] when combination therapy was used for patients with stage II and III NSCLC without *EGFR* or *ALK* alterations. While the oncologic benefits attained from immunotherapy combined with chemotherapy can be cultivated in order to optimize survival outcomes, the effect of immunotherapy on local tissue can potentially render operations more challenging. However, this should not change the trial enrollment or multimodality management as operative mortality and morbidity remained stable but should warrant attention during the peri-operative stage [58]. This aspect of lung cancer therapy continues to evolve, with multiple clinical trials currently ongoing [59].

Immunotherapy and targeted therapy influence advanced NSCLC management. Multiple trials are ongoing and will provide important evidence to add to the patient-centered treatment compendium. Efforts originating from lung cancer mutation consortiums are ongoing and leading the development and characterization of lung cancer allowing for optimization of therapeutic benefit from current agents. It is now becoming standard of care to offer up-front tumor molecular profiling for patients diagnosed with NSCLC with trials [60] investigating its use in informing treatment decisions to specifically select patients with targetable genomic aberrations who may not optimally benefit from immunotherapy (LCMC4 [61], NAUTIKA1 [62], NeoADAURA [63], Dabrafenib/Trametinib Rollover study [64], LIBRETTO-432 [65]).

The incredible evolution of neoadjuvant therapy, with ongoing developments in detecting actionable oncogenic drivers, leads to continued patient-centered neoadjuvant treatment optimization, with surgical resection remaining a meaningful cornerstone of curative management.

### 4.4. Stage III

Stage 3 has been the most controversial lung cancer stage due to its heterogeneity and multiple treatment options yet historically overall poor outcomes. Controversies relate to resectability, single or multiple or “bulky” N2 nodal disease status, contralateral or N3 mediastinal nodal disease, types of neoadjuvant therapy, and the appropriate extent of surgical resection, if any, in this setting. Immunotherapy has been redefining treatment paradigms in this setting and after many years also improving survival (Table 2). Patients with stage III disease benefit from multidisciplinary evaluation with the first decisions being whether the disease is resectable or unresectable. While resectability may be assessed differently by different surgeons, we generally consider patients with stage III NSCLC for operative management if disease control can be achieved via lobectomy and mediastinal node dissection. With the effectiveness of current adjuvant therapies, we do not consider multi-station N2 disease a contraindication. Pneumonectomy should receive individual consideration, especially with N2 disease although N0-1 status is considered for resection. N3 nodal disease remains a contraindication for surgery.

#### 4.4.1. Stage III Resectable Disease

Despite the results originating from a large phase III randomized clinical trial conducted by Albain et al., which showed an insignificant survival benefit associated with resection compared to primary chemo-radiation when patients required a larger resection such as a pneumonectomy [68], lobectomy, coupled with meticulous perioperative care, can provide meaningful outcomes in stage III disease. In highly selected patients, surgical resection plays a significant role in a multimodal therapeutic strategy and is associated with improved overall survival [67] and locoregional recurrence benefit [66]. While selection bias can be a limitation in work published regarding stage III disease, careful designation of patients who will benefit from surgical resection should originate from multidisciplinary meetings and can therefore mirror the inclusion criteria reported in these highly selective clinical trials.

Historically, for patients with stage III disease, with involvement of the ipsilateral mediastinal and/or the subcarinal lymph nodes (N2 disease), whether single-station or multi-station, oncologic benefit was obtained via induction chemotherapy or concurrent neoadjuvant chemoradiotherapy. While it remains the case that patients in this group, with N2 disease, are candidates for induction therapy [69], neoadjuvant chemoradiation can be associated with significant surgical mortality and morbidity [70], and the decision regarding neoadjuvant modality should remain a source of discussion given equivalence in recurrence patterns between neoadjuvant chemoradiation versus neoadjuvant chemotherapy [71]. Patients who require aggressive resections including pneumonectomies should undergo a closely established patient-oriented multidisciplinary discussion regarding goals of care and optimal treatment strategy based on clinicopathologic characteristics. The optimal treatment strategy for this complex group of patients continues to evolve as clinical trials culminate and provide further evidence regarding multimodal approaches such as the INCREASE trial investigating the role of neoadjuvant therapy in resectable and borderline resectable stage III lung cancer patients with tumors larger than 5 cm in size [72,73]. Immunotherapy-containing regimens, with or without chemotherapy, as well as targeted therapies tested or under clinical evaluation in patients with resectable stage III disease as well have been discussed above.

#### 4.4.2. Stage III Unresectable Disease

Patients with stage III disease that is characterized as unresectable, comprising approximately 20% of all cases of lung cancer in the United States [74], will benefit from multimodal therapy, whether for life-prolonging intent, for palliation, or in hopes of converting resectability status.

Historically, the standard of care for this group of patients has involved chemoradiotherapy [73] without induction chemotherapy; however, this continues to be associated with poor overall survival [75,76].

Multiple large trials have investigated the use of immunotherapy or proton therapy in this cohort.

The PACIFIC trial (phase 3), investigating the consolidative use of a PD-L1 inhibitor (durvalumab) for up to 12 months, in patients with stable unresectable stage III disease following chemoradiotherapy, irrespective of PD-L1 expression levels, showed that its use was associated with a prolonged progression-free survival, decreased rate of distant metastasis, and significantly increased time to distant metastasis (23.2 months vs. 14.6 months in placebo) [77,78]. These therapeutic advantages were maintained at a 4-year landmark analysis, with median overall survival in the durvalumab group being 47.5 months compared to 29.1 months in the placebo group (overall survival hazard ratio (HR) = 0.71, progression-free survival (PFS) HR = 0.55) [79].

The benefits of immunotherapy following primary chemoradiation are tenable and resulted in increased consultations for salvage surgical resection in this patient cohort following the development of local or regional recurrence sometimes months to years after the index therapy. While maintenance checkpoint inhibition provides improved outcomes, these sites of recurrence will often have developed significant therapy-related inflammation and fibrosis [80]. Such salvage surgical cases require significant skills and judgment for safe, margin negative resections in order to maintain adequate post-operative mortality and morbidity [81,82].

The landscape of management of unresectable stage III disease is very quickly evolving, and close attention must be paid to guidelines that encompass multimodality and multidisciplinary management of this heterogeneous patient population. Additionally, there are several clinical trials testing or that have evaluated immunotherapy with radiation therapy for patients with unresectable stage III disease, which are beyond the scope of this review focused on resectable disease. 

### 4.5. Stage IV

For patients with stage IV disease, the presence or absence of select actionable genomic alterations and the PD-L1 tumor expression status guide the use of standard of care targeted therapies, immunotherapy and chemotherapy plus immunotherapy (with or without an antiangiogenic agent) [83]. Curative-intent surgery has not been offered for stage IV disease; however, locoregional disease control may have its benefits, especially in the oligometastatic setting. Surgical management for this particular cohort has been shown to provide better overall survival and improvement in disease-free intervals [84]. The extent of resection offered to this patient population is usually limited to lobectomies with mediastinal lymph node resection [85]. A particular principle to consider for this patient population is that while their initial disease stage is metastatic, following responses to therapy, the overall cancer burden may decrease sufficiently to allow for complete visible primary and metastatic disease consolidation and control. 

Specifically, local consolidative therapy with surgery or radiotherapy, in patients with stage IV oligometastatic disease that had not progressed following primary systemic therapy, has shown benefits in progression-free survival and overall survival compared to maintenance therapy or observation [86], which was optimized when adequate consolidative radiation therapy to the primary lesion was achieved at a biologically effective dose [87]. In patients with synchronous multi-site oligometastatic disease, this oncologic benefit was accentuated when comprehensive local consolidative therapy was provided to all sites of disease [88].

Using SABR has also shown oncologic benefit in this population, specifically, in patients with EGFR-negative metastatic NSCLC with up to 5 metastatic sites, with a PFS advantage of 9.7 months compared to 3.5 months in patients managed with maintenance chemotherapy alone [89]. This therapeutic benefit was further established in patients with a controlled primary NSCLC and 1 to 5 metastatic lesions, undergoing SABR in addition to standard of care, with a 5-year OS rate of 42.3% compared to 17.7% in patients receiving standard of care only [90].

In addition, while radiotherapy has a role in the treatment of this cohort, surgical management in patients with operable oligometastatic disease had led to long-term survival or progression-free survival, with similar rates of freedom from locoregional and systemic progression when compared to radiotherapy, accentuating that surgical management of oligometastatic stage IV lung cancer remains a reasonable option in selected patients [84].

Currently there are ongoing clinical trials further exploring multimodal treatment strategies for patients with stage IV disease, such as the LONESTAR trial investigating the role of local consolidation therapy in patients receiving nivolumab and ipilimumab [91], or the NORTHSTAR trial investigating the role of local consolidation therapy in patients with EGFR mutant advanced/metastatic NSCLC receiving osimertinib [92].

## 5. Special Considerations

### 5.1. Pancoast (Superior Sulcus) Tumors

Also known as superior sulcus tumors, Pancoast tumors arise in the lung apex and can invade surrounding soft tissue and will require adequate staging with MRI which will further establish extrapulmonary involvement [93,94]. As suspected, trimodality therapy is a cornerstone in the management of patients with such tumors [95,96]. Following induction neoadjuvant chemoradiotherapy, patients will benefit from surgical resection, regardless of the extent of the procedure or the size of the tumor [97]. High-dose neoadjuvant radiotherapy, when combined with platinum-based chemotherapy, was associated with a high rate of complete pathological response [98], although it also led to an increased rate of post-operative complications [99]. In this cohort, pathological response and nodal status negatively affected overall survival [97]. While this therapeutic model leads to excellent local control with acceptable survival, patients considered for trimodality therapy with high-dose neoadjuvant radiotherapy must continue to be carefully selected [100]. Patients with spinal column invasion will also benefit from a multimodal approach [101], including resection of the tumor and combined chest wall resection with vertebrectomy and spinal reconstruction leading to possible cure, pain control, and preservation of neurological function [102].

### 5.2. Salvage Surgical Management

Patients who do not respond to their primary non-operative therapy might require surgical salvage therapy for persistent or recurrent lung cancer. Patients with early-stage NSCLC who are offered primary SBRT can see a two-year local control rate of 96% [103] while in another series saw a rate closer to 85% [104]. This characterizes a small cohort of patients who might benefit from salvage surgical treatment. Following selection for surgical salvage, patients can benefit from improved overall survival up to 79.5% at 5 years after local recurrence [104].

Similarly, surgical salvage can benefit patients who fail definitive chemoradiotherapy, with a rate of local recurrence up to 35% [105]. The cohort who then undergoes conscientious surgical resection as salvage therapy approximately 7 months following index therapy, can obtain a 2-year survival rate of 46% with 77% of patients receiving complete resections and 25% suffering from post-operative complications [105].

Surgical salvage is feasible and may benefit patients who underwent primary targeted therapy [106] although data regarding outcomes remain scarce.

## 6. The Therapeutic Future of Lung Cancer

Immunotherapy and targeted therapy are rapidly becoming the cornerstones in both operable and metastatic lung cancer. Multiple trials are ongoing and will provide important evidence to add to the patient-centered treatment compendium. Novel biomarker and therapeutic research, however, extends beyond known cancer characteristics. The gut microbiome is an immunological modulator affecting therapeutic responses and efficacy of certain immunotherapy agents such as PD-1, PD-L1, and CTLA-4 inhibitors and has been shown to be associated with positive or negative outcomes from therapy [107] and may serve as a promising modifiable strategy in the treatment of cancer. Recently, microbial species such as *Ruminococcus* and *Akkermansia* have been associated with enhanced major pathologic responses in patients with lung cancer managed with immunotherapy [51]. These exciting results are concordant with findings in patients with melanoma managed with immunotherapy from which differences in anabolic pathways, systemic and antitumor immunity of responding patients were revealed [108], an effect that was then found to be transferable via fecal transplant [108,109], including changes in the tumor microenvironment [109]. However, the gut microbiome is also susceptible to many iatrogenic therapies, and thus many challenges regarding harvesting its benefits remain [110].

The role of radiotherapy in the neoadjuvant setting will also likely be evolving [111], with improvement in diagnostic imaging and targeting techniques and various irradiation modalities including proton beams and stereotactic body radiotherapy providing better localization of radiotherapy while sparing adjacent tissue [73,112], and which has been known to contribute to downstaging [113].

## 7. Conclusions

The field relating to lung cancer management is one of the most exciting there is in surgical oncology, with an incredibly motivated multidisciplinary team relentlessly working to pioneer individualized patient-centered care and tailor current therapies to maximize clinical benefit.

## Figures and Tables

**Table 1:** Selected studies investigating optimal surgical approach in stage I lung cancer.

Investigators	Year	Study Type	Tumor Size	(*n*)	Implications
Altorki et al.	Est. 2024	Randomized Trial	≤2 cm	Est. 701	Active, not yet recruiting trial (accurate 10/2021) NCT00499330 Lobectomy versus sublobar resection for ≤2 cm peripheral lung cancer
Chan et al. [27]	2021	Retrospective Cohort	2.1–3.0 cm	269	No difference in 5-year OS or recurrence between segmentectomy compared to lobectomy
Kamel et al. [5]	2021	Retrospective Cohort	1.5 cm (median)	254	Propensity-matched analysis showed no difference in perioperative complications, overall survival, or cancer-specific survival between lobectomy or sublobar resections
Li et al. [22]	2020	SEER	≤2 cm	5474	Propensity-matched analysis (*n* = 774) showed equivalence of OS and LCSS between lobectomy and segmentectomy
Cao et al. [21]	2018	SEER	≤1 cm	1913	No difference in LCSS between lobectomy, segmentectomy, or wedge resection. OS benefit associated with lobectomy
			1.1–2.0 cm	8761	Similar LCSS associated with lobectomy and segmentectomy, both conferred better LCSS and OS than wedge resection
			2.1–3.0 cm	6145	Lobectomy superior (both OS and LCSS) to wedge resection or segmentectomy. Wedge resection and segmentectomy are similar (OS and LCSS)
Altorki et al. [3]	2018	Randomized Trial	≤2 cm	697	No difference in mortality or morbidity between lobar and sublobar resection Majority of operations performed with MIS (80%), majority of patients ECOG 1 (74%)
Kodama et al. [24]	2016	Retrospective Cohort	≤2 cm	312	Equivalence in LRFS between lobectomy and segmentectomy, with OS benefit associated in lobectomy in full-cohort analysis. Propensity-matched analysis (*n* = 138) showed equivalence in OS and LRFS
Landreneau et al. [26]	2014	Retrospective Cohort	2.2 cm (mean)	624	No significant difference in OS or Recurrence between lobectomy and segmentectomy
Altorki et al. [4]	2014	Retrospective Cohort	≤3 cm	337	No difference in survival between lobar and sublobar resection. Subgroup analysis of tumor size ≤2 cm showed survival benefit associated with sublobar resection (*n* = 306)
Ginsberg et al. [2]	1995	Randomized Trial	≤3 cm	247	No difference in mortality or morbidity between lobar and limited resection. A 75% increase in recurrence rate in limited resection, 30% increase in overall death rate.

Abbreviations: (*n*): number of patients included in study, Est.: estimated, OS: overall survival, SEER: Surveillance, Epidemiology, and End Results database, LCSS: lung-cancer-specific survival, ECOG: Eastern Cooperative Oncology Group, LRFS: locoregional recurrence-free survival.

**Table 2:** Selected studies investigating multimodal management of stage III lung cancer.

Investigators	Year	Study Type	Inclusion	(*n*)	Implications
Spicer et al. [53] (Checkmate816)	2021	Randomized Trial	Stage IB-IIIA	358	Addition of nivolumab to neoadjuvant chemotherapy led to increased depth of pathological response. Majority of patients stage IIIA (63%)
Provencio et al. [48] (NADIM)	2020	Randomized Trial	Resectable Stage III	46	Patients with resectable stage III disease should receive neoadjuvant nivolumab with platinum-based chemotherapy prior to resection. Majority of patients T1N2 (33%) and T3N2 (28%)
Antonia et al. [50,51] (PACIFIC)	2017–2018	Randomized Trial	Unresectable Stage III	713	Consolidation therapy with durvalumab associated with better OS and PFS compared to placebo, regardless of PD-L1 expression
Bott et al. [66]	2015	NCDB	T4N2 or Any N3	9173	Surgical resection as part of multimodal treatment was associated with improved OS. Propensity-matched analysis confirmed results (*n* = 1262)
Albain et al. [67]	2009	Randomized Trial	T1-3pN2	202	No difference in OS, better PFS in group receiving surgical resection as part of multimodal treatment. Majority of patients T2 (63%), cN1 (76%)

Abbreviations: (*n*): number of patients included in study, OS: overall survival, PFS: progression-free survival, pN2: pathologic N2 status, cN1: clinical N1 status.
